# Stable Thermally-Modulated Nanodroplet Ultrasound Contrast Agents

**DOI:** 10.3390/nano11092225

**Published:** 2021-08-29

**Authors:** Anastasiia Vasiukhina, Javad Eshraghi, Adib Ahmadzadegan, Craig J. Goergen, Pavlos P. Vlachos, Luis Solorio

**Affiliations:** 1 Weldon School of Biomedical Engineering, Purdue University, West Lafayette, IN 47907, USA; avasiukh@purdue.edu (A.V.); cgoergen@purdue.edu (C.J.G.); 2 School of Mechanical Engineering, Purdue University, West Lafayette, IN 47907, USA; jeshragh@purdue.edu (J.E.); aahmadza@purdue.edu (A.A.); 3 Center for Cancer Research, Purdue University, West Lafayette, IN 47907, USA

**Keywords:** ultrasound, contrast agent, nanodroplet, perfluorocarbon, nanotechnology, biosensor, phase-shift, nanoemulsion, thermally-responsive

## Abstract

Liquid perfluorocarbon-based nanodroplets are stable enough to be used in extravascular imaging, but provide limited contrast enhancement due to their small size, incompressible core, and small acoustic impedance mismatch with biological fluids. Here we show a novel approach to overcoming this limitation by using a heating–cooling cycle, which we will refer to as thermal modulation (TM), to induce echogenicity of otherwise stable but poorly echogenic nanodroplets without triggering a transient phase shift. We apply thermal modulation to high-boiling point tetradecafluorohexane (TDFH) nanodroplets stabilized with a bovine serum albumin (BSA) shell. BSA-TDFH nanodroplets with an average diameter under 300 nanometers showed an 11.9 ± 5.4 mean fold increase in echogenicity on the B-mode and a 13.9 ± 6.9 increase on the nonlinear contrast (NLC) mode after thermal modulation. Once activated, the particles maintained their enhanced echogenicity (*p* < 0.001) for at least 13 h while retaining their nanoscale size. Our data indicate that thermally modulated nanodroplets can potentially serve as theranostic agents or sensors for various applications of contrast-enhanced ultrasound.

## 1. Introduction

Ultrasound (US) is one of the most widely used clinical imaging modalities. It is used in cardiology and vascular imaging, urology, gynecology, obstetrics, as well as in general abdominal imaging [1]. US imaging is portable, non-ionizing, and has a high spatial and temporal resolution [2]. One limitation of ultrasound is its poor contrast resolution, which often makes the differentiation between the blood pool and adjacent soft tissues difficult. Contrast resolution can be enhanced by using contrast agents to facilitate the improved visualization of anatomical structures and more accurate diagnosis. Moreover, ultrasound contrast agents (UCA) can also be used to provide therapeutic benefits in US-mediated thrombolysis [3], tumor ablation [4], sonoporation [5], and drug and gene delivery [6].

Currently, all commercially available UCAs are fluorocarbon gas-filled microbubbles stabilized by a lipid or protein shell that are administered into the circulation [7]. Microbubbles intensify acoustic backscattering due to a significant impedance mismatch between the gas in their core and the surrounding aqueous medium, as well as oscillatory responses in a US field [8]. Currently, commercialized microbubbles have circulation times of 5–10 min, and are typically larger than 2 μm in diameter [9]. The large hydrodynamic diameters and limited stability of these agents restrict them to intravascular and cardiac applications. In order to expand the application of UCAs beyond vascular and cardiac imaging, gas-based nanobubbles (NB) have been developed [10]. Several of the nanobubble formulations created to date exhibit echogenicity comparable to commercially available microscale bubbles [11,12,13]. The echogenicity signal stability of NBs typically ranges from several min [14,15] to an hour [16], although Perera et al. created a nanobubble formulation that retained echogenic signal for 24 h [17]. The nanobubble’s stability is impacted by the stabilizing shell’s composition. Shell materials that reduce the surface tension of the membrane during expansion and contraction result in more stable nanobubbles [18]. Additionally, shell structures that can resist deformation under prolonged US exposure and flow conditions also improve nanobubble stability [19].

The stability of nanoscale shell-encapsulated contrast agents can also be increased by substituting the gas core with a liquid perfluorocarbon (PFC). Liquid PFC emulsions remain stable in circulation for up to several days [20]. The safety profile of liquid PFCs is well established, as they have been used in liquid-assisted ventilation [21] and as artificial red blood cell substitutes [22]. However, the echogenicity of nanodroplets is very low due to the small acoustic impedance mismatch between the liquid PFC core of the particles and the surrounding tissues or biological fluids [23]. Additionally, the incompressibility of the liquid core further contributes to the low echogenicity of the nanodroplets [2]. However, when exposed to acoustic energy of sufficient amplitude, non-echogenic liquid PFC-based nanodroplets become echogenic gas microbubbles via acoustic droplet vaporization (ADV) [24]. ADV is typically performed on nanodroplets composed of PFCs with boiling points close to body temperature, such as dodecafluoropentane and perfluorohexane, although phase-change agents containing low-boiling point PFCs (for example, octafluoropropane) have been created as well [25].

Working with acoustically-triggered phase-change nanodroplets is subject to many challenges, including the need for high vaporization energy, nonuniformity of activation in polydisperse particle samples, and carefully balancing droplet vaporization thresholds and thermal stability in various applications [26]. Similar to ADV induced by ultrasound, thermal energy also triggers the conversion of the liquid PFC core into a gas phase, as evidenced by the spontaneous vaporization events reported for PFC droplets [27]. When PFC droplets phase-transition, they undergo volumetric expansion with an average radial expansion ratio of 5 [28]. The average in vivo half-life of PFC bubbles after ADV, according to a study performed using a canine model, is 8 min [29].

Herein, we introduce a novel method for increasing nanodroplet echogenicity that preserves the nanoscale size and stability of the droplets. We discovered that liquid TDFH nanodroplets, stabilized with a BSA shell, exhibit a thermal response that increases their echogenicity without inducing a transient and complete phase shift. This thermal response is elicited when nanodroplets are subjected to a heating–cooling cycle, a process that we refer to as thermal modulation (TM). In this study, TM was applied to BSA-TDFH nanodroplets that were prepared and characterized in terms of structural property, size, echogenicity, stability, and thermal responsiveness.

## 2. Materials and Methods

### 2.1. Nanodroplet Formulation and Characterization

#### 2.1.1. BSA-Shelled Tetradecafluorohexane Nanodroplet Formulation

BSA-TDFH nanodroplets were prepared fresh on the day of the experiment by combining 250 μL of liquid TDFH (Sigma Aldrich, St. Louis, MO, USA) at −20 °C with 850 μL of a 1.4% solution of BSA (Rockland antibodies and assays) in 2 mL glass scintillation vials. The vials were then capped and sealed with Parafilm, and the air was removed using a 10 mL syringe. Each vial was then agitated on a VialMix shaker (Lantheus Medical Imaging, N. Billerica, MA, USA) for 135 s to form the nanodroplets. The droplets were then stored at 4 °C for 30 min before use.

Sample formulations were then prepared by diluting the stock solution to the desired concentration with 1X PBS. Then, the sample was centrifuged at 500× *g* rcf for 5 min to isolate nano-sized droplets from an initially polydisperse particle population.

#### 2.1.2. Nanodroplet Characterization

Transmission electron microscopy (TEM) was used to provide information about particle morphology. The mean hydrodynamic diameter and zeta potential were then measured using dynamic light scattering (DLS). Confocal imaging was used to directly visualize the nanodroplets before and after thermal modulation. Before obtaining the TEM and confocal images, as well as DLS size measurements, sample formulations were diluted 1:167 with 1X PBS. For DLS zeta potential measurements, nanodroplet samples were diluted 1:167 with 1 mM KCl instead of PBS. Diluted particle concentrations were used to minimize multiple scattering and particle–particle interactions during DLS measurements, and to limit the fluorescence field saturation during confocal imaging.

To prepare samples for TEM, first, the TEM grids (carbon film only on 200 mesh, copper; Ted Pella) were glow-discharged for 30 s before sample application. Next, 3 μL aliquots of nanodroplet samples were applied to the grids. The grids were then blotted from the side with filter paper to remove excess liquid, and then placed on a sample holder. Images were acquired using a Gatan US1000 2K CCD camera on an FEI Tecnai T20 electron microscope equipped with a LaB6 source and operating at 200 kV.

The average hydrodynamic diameter and zeta potential of BSA-TDFH nanodroplets were measured using DLS on a Zetasizer Nano ZS90 (Malvern Panalytical, Malvern, UK) at 22 °C.

The nanodroplets were prepared with fluorescent BSA (Alexa Fluor 594 conjugate) for confocal imaging. A Zeiss LSM 880 confocal microscope (Carl Zeiss Microscopy, LLC, Jena, Germany) was used to image the nanodroplets in a microfluidic slide (µ-Slide VI 0.4, Ibidi) with 400 µm channel height. The channel was viewed through a 63x oil objective lens (numerical aperture NA = 1.4) and illuminated by an Argon laser (561 nm wavelength). The nanodroplets were imaged before thermal modulation at 25 °C, and after thermal modulation at 37 °C. The images were obtained at five different Z locations inside the microfluidic channel: Z = 0 µm (at the level of the glass), 20 µm, 40 µm, 100 µm, and 200 µm above the glass. A time series of 1000 cycles was recorded at each position, with a pixel dwell time of 0.85 µsec and a scan time of 65 msec. The pixel size was set to 0.11 µm. A heated confocal stage was used to maintain the particles at 37 °C after thermal modulation.

The confocal image series were then processed using an image-based probability estimation of displacement (iPED) method to find the average diffusion coefficient of the droplets in the solution [30]. The Stokes–Einstein equation was used to determine the size of the particles based on the measured diffusion coefficient, solution viscosity, and temperature [31]. The average radii of the nanodroplets were found at each scanning height. The average size of the particles within each sample was found by taking the weighted average size of all scanned levels using the nanodroplet counts at each level. The number of nanodroplets at each level was found using an adapted version of mutual information [32]. Mutual information (MI) is a method for counting moving objects in images based on the cross-correlation of consecutive frames. MI finds the number of particles from the ratio of the height of the cross-correlation peak to the height of the auto-correlation peak for one particle. The MI method is designed for cases where all the objects move with the same velocity, and does not account for objects’ random displacement. However, nanodrops undergo a random (Brownian) motion. Therefore, instead of using the height of the peaks, we used the energy of correlations, that is, the volume under the cross-correlation plane and autocorrelation of a single nanodroplet. The energy of the correlation is agnostic to the displacement patterns, and can provide a reliable measurement of object count.

### 2.2. Echogenicity Characterization

To analyze the echogenicity and stability of the nanodroplets, a diluted nanodroplet solution was added to a 150 mL glass beaker and stirred continuously at 150 rpm on a temperature-controlled hotplate (Thermo Fisher Scientific, Waltham, MA, USA) to agitate the nanodroplets. The particles were diluted 1:167 with 1X PBS for the repeatability study, and 1:64 for the stability and temperature sweep studies. The nanodroplets were imaged using a Vevo 3100 (FUJIFILM VisualSonics, Toronto, CAN) preclinical ultrasound imaging system with an MX250 linear array transducer with a center frequency of 21 MHz and axial resolution of 75 μm. Images were acquired using both B-mode and nonlinear contrast (NLC) mode. The following image acquisition settings were used for the studies: a frequency of 18 MHz, 75% power, a 40 dB dynamic range, a 22 frame per second (fps) acquisition frame rate, a 30 dB gain, and a 30 dB contrast gain. The acquisition depth was set to 9 mm and the focal depth was set to 5 mm. Following each study, the data were exported as MP4 files for image processing and statistical analysis.

Ultrasound image series were converted to grayscale images, and the boundaries of each nanodrop were segmented using an in-house developed MATLAB (MathWorks, Natick, MA, USA) code [33]. A three-point Gaussian curve was fitted to the intensity values of each identified nanodrop to find the actual maximum intensity. Next, the maximum intensity of each nanodrop was multiplied by the area of the corresponding nanodrop to get the nanodrop weighted intensity. The intensity level of each frame was then defined as the summation of the nanodrop weighted intensity over all the identified nanodrops. The intensity level was then calculated for all the frames to obtain the intensity level signal for each image series.

#### 2.2.1. Repeatability of Thermal Modulation

We used TM to evaluate the effect of heat on TDFH nanodroplet echogenicity. First, we heated the dilute nanodroplet solution from room temperature to a setpoint of 42 ± 2 °C. The solution was then allowed to cool to a setpoint of 37 ± 2 °C (*n* = 15). This temperature range is sufficiently below the vaporization threshold of pure TDFH (56 °C). An image series of the droplets (350 frames) was recorded before thermal modulation and after, and the images were processed to quantify the effects of thermal modulation. The data were plotted as mean signal intensity values after TM normalized by the average signal intensity before modulation. The fold-increase in echogenicity post-treatment was reported as an overall mean ± standard deviation (STD).

#### 2.2.2. Stability

The echogenicity signal stability of the nanodroplets after undergoing TM was tracked for 13 h. A total of 350 frames were recorded for each sample (*n* = 5) at the designated time points (t = 0, 1, 2, 3, and 13 h), and the mean signal intensity normalized by the average intensity at t = 0 h was plotted as a function of time.

#### 2.2.3. Temperature Sweep

The effect of temperature on nanodroplet echogenicity was investigated by heating the nanodroplet solution from room temperature to 44 °C, and subsequently cooling back to room temperature (*n* = 5). Images were acquired during the heating and cooling parts of the thermal cycle, at 25, 28, 31, 34, 37, 40, 43, and 44 °C. The mean signal intensity normalized by the average intensity at 25 °C was plotted as a function of temperature.

### 2.3. Statistical Analysis

All data are presented as mean ± STD unless stated otherwise. A minimum of 5 replicates were performed for each study. Statistical analysis was performed using Minitab 19 software. Paired t-tests were used to determine the significance between experimental groups. The Tukey test was performed to derive multiple pairwise statistical comparisons in the stability study.

## 3. Results

### 3.1. Nanodroplet Size Distribution and Structural Property

**Figure 1:**
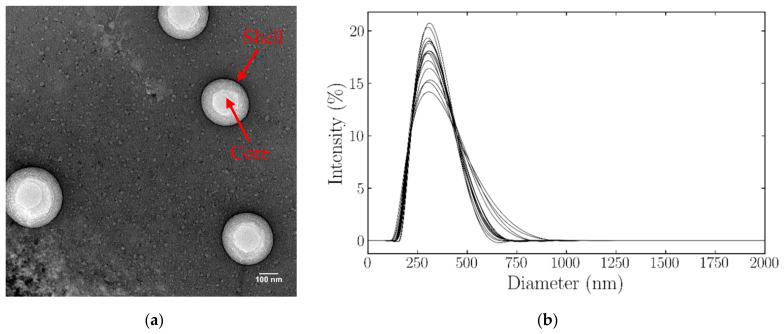
Internal structure and size distribution of BSA-shelled TDFH nanodroplets (**a**) The morphology of BSA-TDFH nanodroplets visualized on the TEM. Scale bar = 100 nm. (**b**) Size distribution of nanodroplets obtained by DLS measurements.

TEM was used to show the structure of the particles. TEM images (Figure 1a) revealed spherical particles with a distinguishable core and surrounding shell.

The hydrodynamic diameter of the nanodroplets was then measured using DLS (Figure 1b). The mean hydrodynamic diameter of the particles was found to be 299.0 ± 3.356 nm with a polydispersity of 0.092 ± 0.030 (*n* = 5). The mean nanodroplet zeta potential was −33.1 ± 1.06 mV (*n* = 5).

### 3.2. In Vitro Thermal Modulation: Contrast Enhancement and Repeatability

**Figure 2:**
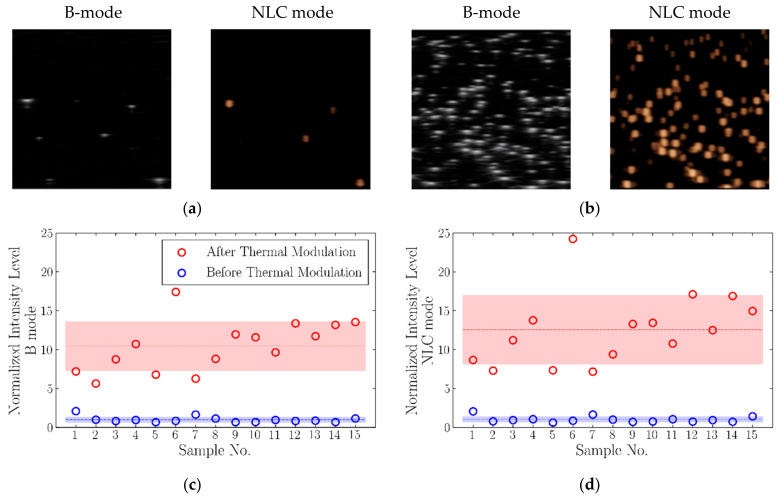
Analysis of nanodroplet echogenicity before and after thermal modulation. Representative ultrasound images of nanodroplets on the B-mode and the NLC mode (**a**) before and (**b**) after thermal modulation. (**c**) Nanodroplet signal intensity on the B-mode before and after thermal modulation. The data were normalized by the grand mean of signal intensity before the thermal cycle. (**d**) Nanodroplet signal intensity on the NLC mode before and after thermal modulation, normalized by the grand mean of signal intensity before treatment.

The ultrasound signal intensity on the B-mode and the nonlinear contrast (NLC) mode before and after TM was quantified to determine the change in signal for the nanodroplets that underwent a thermal cycle (Figure 2c,d). The thermal modulation experiment was repeated *n* = 15 times, and the ultrasound images were acquired before TM at room temperature and after modulation at 37 °C.

**Figure 3:**
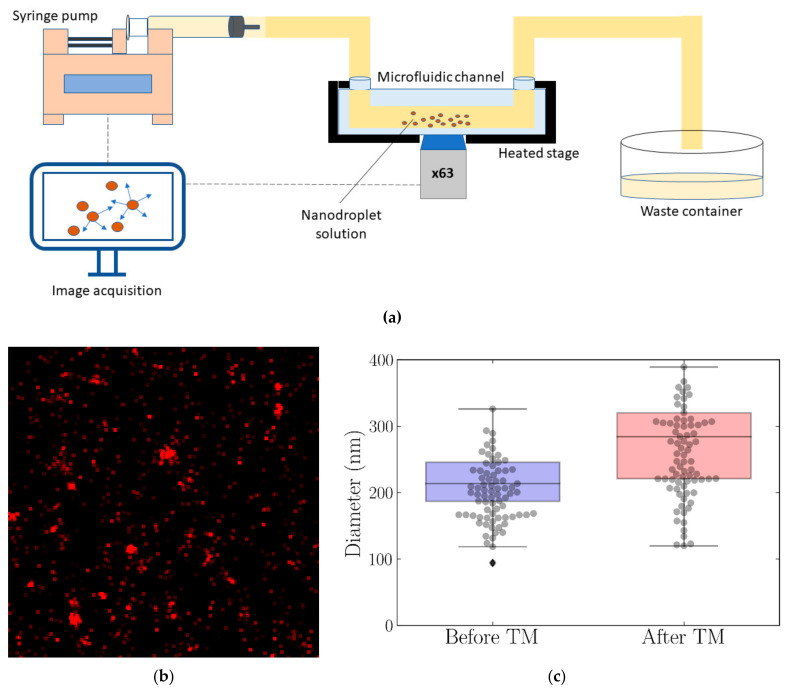
Size distribution of the nanodroplets obtained by confocal imaging in combination with the iPED method. (**a**) A schematic of the experimental setup used to acquire confocal microscope images of the BSA-TDFH nanodroplets in the microfluidic channel. (**b**) A representative confocal image used for iPED analysis. (**c**) Nanodroplet size distribution before and after thermal modulation.

Thermal modulation significantly increased the echogenicity of the nanodroplets (*p* < 0.001). The mean fold increase in nanodroplet echogenicity after the thermal cycle was 11.9 ± 5.4 on the B-mode and 13.9 ± 6.9 on the NLC mode. To determine whether such a significant increase in echogenicity could be attributed to the phase transition of the nanodroplets’ TDFH core from liquid to gas and the particles’ conversion from nanodroplets to microbubbles, the confocal images of nanodroplets in a microfluidic device were acquired as a time series before and after modulation (*n* = 5), and the size of particles was determined using the iPED method (Figure 3). The data showed that after the thermal cycle, a significant increase (*p* = 0.006) in the average diameters of nanodroplets occurred, with a mean diameter of 216.0 ± 10.5 nm before TM and 272.6 ± 27.4 nm after TM.

### 3.3. Nanodroplet Stability after Heat Modulation

**Figure 4:**
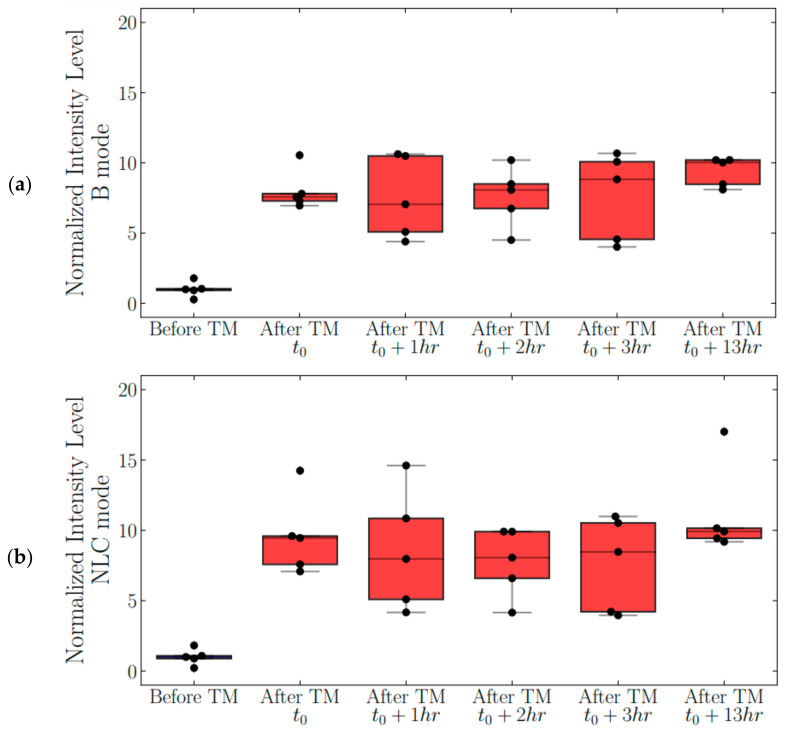
Analysis of nanodroplet stability. (**a**) Ultrasound signal intensity on the B-mode as a function of time for a 13 h period (*n* = 5). (**b**) Ultrasound signal intensity on the NLC mode as a function of time for 13 h (*n* = 5).

The echogenicity of thermally modulated TDFH nanodroplets was monitored for 13 h post-heat activation (Figure 4a,b). The Tukey test for all pairs showed that the mean normalized signal intensity levels at all time points after TM were significantly different from the mean intensity level before modulation, but not statistically different from each other, indicating that the particles were stable once activated for at least 13 h.

### 3.4. Nanodroplet Echogenicity as a Function of Temperature

**Figure 5:**
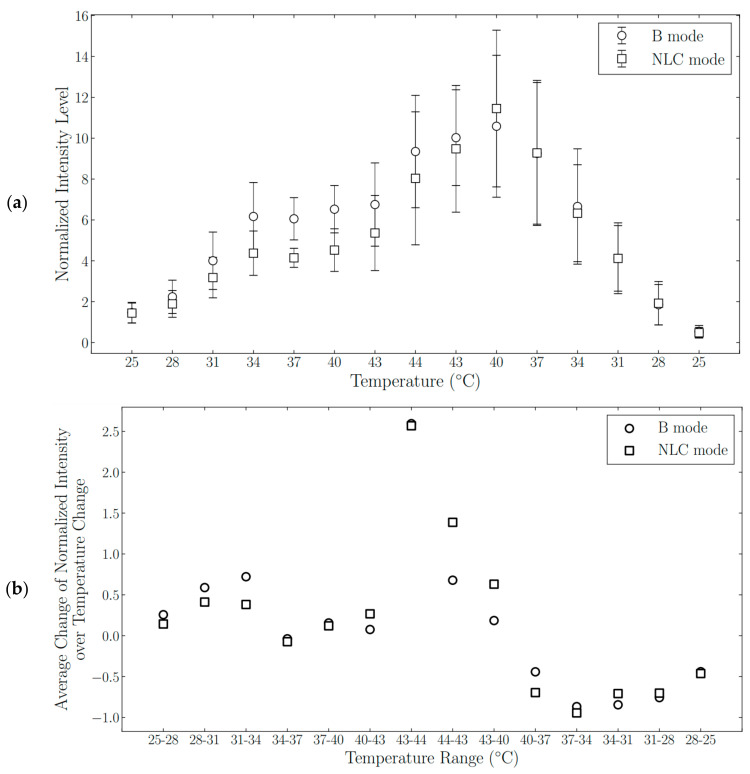
Analysis of thermally responsive nanodroplet behavior. (**a**) Nanodroplet signal intensity as a function of temperature in the B-mode and the NLC mode (*n* = 5). The data were normalized by the mean signal intensity at 25 °C. (**b**) The mean rate of change of nanodroplet echogenicity with respect to temperature plotted over selected temperature intervals.

As shown in Figure 5a, nanodroplet echogenicity displayed a thermally dependent behavior over a range of temperatures. When the solution temperature was increased from 25 °C to 34 °C, the average rate of change in nanodroplet echogenicity was positive (Figure 5b), and the particle signal intensity was increasing. When the solution temperature was further increased from 34 °C to 43 °C, the derivative was near zero, and the nanodroplet echogenicity remained relatively constant. Upon further heating from 43 °C to 44 °C, the nanoparticle signal intensity increased rapidly, as evidenced by the spike in the graph of the mean rate of change in nanodroplet echogenicity. After the solution reached the peak temperature of 44 °C and began to cool down, the derivative of nanodroplet echogenicity with respect to temperature continued to be positive over the 44 °C to 43 °C range. Then, the derivative changed from being positive in the interval from 43°C to 40 °C to being negative in the interval from 40 °C to 37 °C. As a result, the maximum nanoparticle echogenicity during the thermal cycle was recorded not at the peak temperature of 44 °C, but at 40 °C, during the cooling portion of the cycle. The mean rate of change of nanodroplet signal intensity remained negative in the temperature interval from 37 °C to 25 °C, and the nanodroplet echogenicity decreased to the baseline value when the solution was cooled back to 25 °C. Figure S1 highlights that nanodroplets had higher signal intensity at 37 °C during the cooling period compared to when they first reached that temperature in the heating part of the thermal sweep. Therefore, a higher echogenicity of BSA-TDFH nanodroplets was induced with TM compared to direct heating to 37 °C.

## 4. Discussion

PFC nanodroplets naturally exhibit negligible US contrast properties. This study discovered that BSA-TDFH nanodroplets have a temperature-sensitive echogenic behavior that is independent of transient droplet vaporization, and we showed that thermal modulation could be used as a novel method to induce echogenicity to PFC nanodroplets.

We showed that the echogenicity of TDFH nanodroplets was enhanced without a transient phase change of the liquid core by thermal modulation (subjecting the particle solution to a heating and cooling cycle). The method proved to be effective as it induced a mean fold increase of 11.9 ± 5.4 in nanodroplet signal intensity on the B-mode and 13.9 ± 6.9 on the NLC mode. The average size of the nanodroplets increased after TM, but remained below 300 nm, suggesting that the enhanced echogenicity was not caused by the significant volumetric expansion of the particles, as is typical for cases of ADV. This was expected, as the heating range for heat modulation experiments (room temperature −44 °C) was far below the vaporization temperature of pure liquid TDFH (56 °C). Although we used a heated confocal stage to keep thermally modulated nanodroplets at 37 °C during image acquisition, it is possible that the actual temperature inside the chamber was slightly different from the setpoint, which would have affected the size calculations. Minor changes in the solution viscosity with varying temperature could have played a role as well.

The echogenicity of BSA-shelled TDFH nanodroplets was temperature-sensitive, and the signal intensity generally increased with increasing temperature and declined with decreasing temperature. Three separate regions of echogenic behavior could be differentiated in the heating interval of the thermal cycle: initial increase in nanodroplet signal intensity followed by a plateau, then another rapid increase. The shape of nanodroplet echogenicity versus the temperature graph in that region closely resembled that of a typical heating curve of a substance, where plateaus indicate a phase change. Therefore, it is possible that in the interval from 34 °C to 43 °C, during heating, the liquid nanodroplet core underwent partial phase transition, which led to a rapid echogenicity increase when more heat was added to the system. This could help explain the observed increase in average nanodroplet diameter after TM, as well as the change in echogenicity as a function of temperature on the NLC mode, which detects harmonic signals resulting from nonlinear oscillations characteristic of gas-based particles under insonation. After the particle solution reached the peak temperature, the rate of change of nanodroplet echogenicity with respect to temperature remained positive until the solution was cooled to 40 °C. Interestingly, the nanoparticles at 37 °C during the cooling part of the thermal cycle had a stronger signal compared to when they first reached that same temperature during the heating portion of the thermal sweep. Therefore, thermal modulation yielded more echogenic particles than direct heating to 37 °C.

After undergoing thermal modulation, the nanodroplets exhibited echogenic stability in vitro and retained their enhanced echogenicity over the course of 13 h. Fluctuations in signal intensity were observed over time, likely due to the solution’s temperature oscillating around the setpoint and thus affecting the echogenicity of the particles. No decay in signal intensity was observed throughout the stability study.

Our results suggest that thermal modulation is a novel method for inducing echogenicity in BSA-TDFH nanodroplets. In contrast to ADV, thermal modulation preserves the nanoscale size of the particles and their echogenic stability. The application of PFC nanodroplets in their unaltered state in US imaging and US-mediated therapies has been limited due to their weak echogenic properties. However, the use of thermal modulation has the enabling potential to expand the range of applications of stable nanodroplets in diagnostic and therapeutic ultrasound.

One limitation of this study is that the underlying principle of thermally induced nanodroplet echogenicity remains unclear. The fact that nanodroplets exhibit thermally dependent behavior in a range of temperatures from 25 °C to 44 °C is very interesting, because our system does not seem to follow the anticipated droplet physics. Droplets/bubbles in solution experience both ambient pressure and an additional pressure called Laplace pressure, which arises from interfacial surface tension effects and is inversely proportional to the radius of the particles [34]. The Laplace pressure equation is expressed as

$$\Delta P=P_{inside}-P_{outside}=\frac{2\sigma }{r}$$

where *P_inside_* is the pressure inside the particle, *P_outside_* is the ambient pressure of the surrounding fluid, r is the particle radius, and σ is the surface tension. According to this equation, smaller droplets experience greater pressure and, therefore, a significant increase in vaporization temperature, as predicted by the Antoine equation [35]. The Antoine equation is expressed as

$$\log_{10}P=A-\frac{B}{T+C}$$

where *P* is pressure, *T* is temperature, and *A*, *B*, and *C* are substance-specific coefficients. According to this equation, the predicted vaporization temperature of perfluorohexane droplets that are 300 nm in diameter is approximately 135 °C [36]. Since our system does not seem to follow the anticipated nanodroplet physics, more work is needed to unify theoretical predictions with the experimental outcomes. Unfortunately, currently available imaging techniques and analytical methods are not ideal for studying the processes occurring at the nanoscale, which has limited our ability to investigate any changes taking place in the shell and the core of the nanodroplets as they undergo thermal modulation. Understanding what causes the echogenicity to increase would significantly help us in optimizing this technology. Additionally, an animal study will be necessary to confirm the contrast-enhancing ability of the nanodroplets after thermal modulation, since introduction into the blood pool could have a significant effect on particle performance. Future studies will focus on investigating the echogenic properties of thermally modulated BSA-TDFH nanodroplets in vivo. We will also explore the effect of various shell–core material compositions on nanodroplet signal intensity after thermal modulation. For example, substituting a stiffer protein shell with a more elastic lipid shell may help enhance the acoustic properties of the nanodroplets [37,38]. We also plan to investigate the potential role of nucleate boiling in nanodroplet echogenicity enhancement after thermal modulation [39]. Finally, we will attempt to improve our thermal modulation method by studying the effects of different peak temperatures and heating/cooling rates on the echogenicity of the nanodroplets.

## 5. Conclusions

We report here that the echogenicity of protein-shelled TDFH nanodroplets exhibits thermal responsiveness over a temperature range below the phase transition temperature of pure TDFH. Hence, we developed a novel thermal modulation method for the controlled, vaporization-independent activation of the liquid-based ultrasound contrast agent. This simple yet effective method induced echogenicity in traditionally poorly echogenic PFC-based nanodroplets, while preserving their nanoscale size and stability. Thermally-modulated BSA-TDFH nanodroplets showed more than a tenfold increase in echogenicity, on average, and maintained the enhanced signal for 13 h.

## Data Availability

The data will be made available upon request.

## Supplementary Materials

The following are available online at https://www.mdpi.com/article/10.3390/nano11092225/s1, Figure S1: The ratio of nanodroplet signal intensity during cooling and heating portions of the thermal cycle for selected temperature points.
