# Supplementary material for: Stable Thermally-Modulated Nanodroplet Ultrasound Contrast Agents
Source: Nanomaterials (Basel). 2021 Aug 29;11(9):2225. doi: 10.3390/nano11092225 (PMC8469504; doi:10.3390/nano11092225)
Supplement: Supplementary file 1 [file nanomaterials-11-02225-s001.zip › nanomaterials-1305657-supplementary.pdf]

nanomaterials

Supplementary Materials

Stable Thermally-Modulated Nanodroplet Ultrasound Contrast Agents

Anastasiia Vasiukhina [1], Javad Eshraghi [2], Adib Ahmadzadegan [2], Craig J. Goergen [1,3], Pavlos P. Vlachos [2,*] and Luis Solorio [1,3,*]

1 Weldon School of Biomedical Engineering, Purdue University, West Lafayette, IN 47907, USA; avasiukh@purdue.edu (A.V.); cgoergen@purdue.edu (C.J.G.)
2 School of Mechanical Engineering, Purdue University, West Lafayette, IN 47907, USA; jeshragh@purdue.edu (J.E.); aahmadza@purdue.edu (A.A.); pvlachos@purdue.edu (P.P.V.)
3 Center for Cancer Research, Purdue University, West Lafayette, IN 47907, USA
* Correspondence: lsolorio@purdue.edu

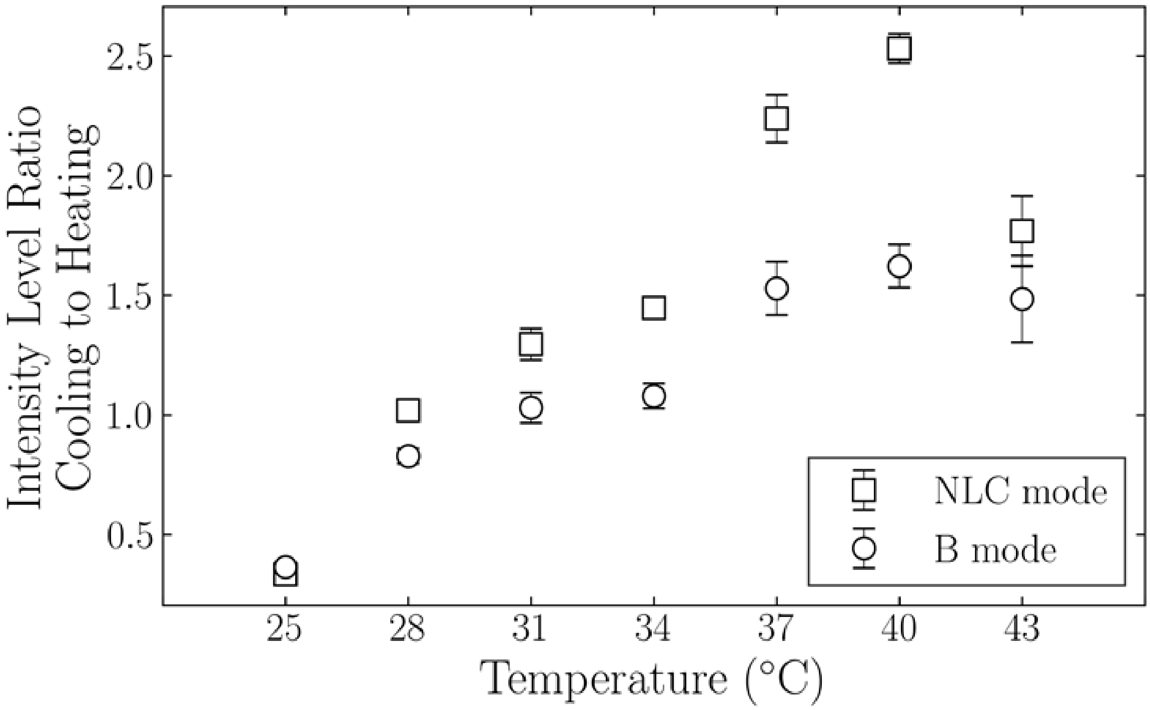

Figure S1. The ratio of nanodroplet signal intensity during cooling and heating portions of the thermal cycle for selected temperature points.
